# Recent Advances in Small Peptides of Marine Origin in Cancer Therapy

**DOI:** 10.3390/md19020115

**Published:** 2021-02-19

**Authors:** Qi-Ting Zhang, Ze-Dong Liu, Ze Wang, Tao Wang, Nan Wang, Ning Wang, Bin Zhang, Yu-Fen Zhao

**Affiliations:** 1 Institute of Drug Discovery Technology, Ningbo University, Ningbo 315211, China; 1811075025@nbu.edu.cn (Q.-T.Z.); 1911074031@nbu.edu.cn (T.W.); zhaoyufen@nbu.edu.cn (Y.-F.Z.); 2 Li Dak Sum Yip Yio Chin Kenneth Li Marine Biopharmaceutical Research Center, Department of Marine Pharmacy, College of Food and Pharmaceutical Sciences, Ningbo University, Ningbo 315800, China; 1911085033@nbu.edu.cn (Z.-D.L.); 2011085073@nbu.edu.cn (Z.W.); 3 Quality Assurance Department, Shenzhen Kivita Innovative Drug Discovery Institute, Shenzhen 518057, China; nan.wang@szkivita.com

**Keywords:** marine organism, anticancer medicine, small peptide, liner peptide, cyclic peptide

## Abstract

Cancer is one of the leading causes of death in the world, and antineoplastic drug research continues to be a major field in medicine development. The marine milieu has thousands of biological species that are a valuable source of novel functional proteins and peptides, which have been used in the treatment of many diseases, including cancer. In contrast with proteins and polypeptides, small peptides (with a molecular weight of less than 1000 Da) have overwhelming advantages, such as preferential and fast absorption, which can decrease the burden on human gastrointestinal function. Besides, these peptides are only connected by a few peptide bonds, and their small molecular weight makes it easy to modify and synthesize them. Specifically, small peptides can deliver nutrients and drugs to cells and tissues in the body. These characteristics make them stand out in relation to targeted drug therapy. Nowadays, the anticancer mechanisms of the small marine peptides are still largely not well understood; however, several marine peptides have been applied in preclinical treatment. This paper highlights the anticancer linear and cyclic small peptides in marine resources and presents a review of peptides and the derivatives and their mechanisms.

## 1. Introduction

Oceans cover about 70% of the earth’s surface and 95% of the biosphere. Water was the cradle of the earliest living organisms, containing approximately 75% of all living organisms. The marine environment offers a rich source of natural products with potential therapeutic applications. More than 1 million marine invertebrates and more than 25,000 species of fish have been discovered, and some of these have been shown to contain natural products with potential biological activity [1,2]. In recent years, marine microorganisms, have also been regarded as a valuable source of bioactive compounds, with the advantages of easy cultivation and good compound extraction repeatability [3]. More than 10,000 bioactive molecules that have been isolated from marine organisms, and several have been found to possess anticancer activity [4]. Most of these natural products with anticancer activity originate from microorganisms (bacteria, fungi, protozoa, viruses, and chromista), plantae (flowering plants like mangroves and macroalgae), and animalia (invertebrates such as sponges, tunicates, and vertebrates such as fish and whale), etc.

Cancer is one of the leading causes of death in the world. An estimated 9.6 million people died of cancer in 2018 [5]. Almost 1 in 6 people die of cancer globally. With the application of new theories, modern technologies, and new drugs in basic tumor research and clinical treatment, the rising trend in tumor death in many countries has been effectively controlled [5]. Chemotherapy is part of the major categories of medical oncology. Despite these successes, chemotherapy’s lingering toxic side-effects are still a primary cause of morbidity and mortality in cancer survivors [6]. As for traditional chemotherapy drugs, most of these inhibit tumor cell proliferation by acting on the DNA synthesis and the replication of tumor cells, which have been shown to be effective but at the price of high toxicity due to a lack of selectivity. Nowadays, there are novel molecular methods for treatment using cancer drugs, including target therapy by cell surface receptors, immune-directed therapy, therapeutic vaccines, and antibody–drug conjugates (ADCs) [7,8]. According to the 2020 ASCO’s Annual Report, a large number of innovative drugs, which can be categorized into targeted drugs and immune drugs, have entered trials and clinical trials [9]. Targeted medicine can restrain tumor cell growth by blocking the signal transduction, but the recurrence rate is extremely high [10]. Antibody–drug conjugates have the potential for increased tumor penetration and drug resistance. It has been demonstrated that a knottin peptide–drug conjugate (KDC) can selectively deliver gemcitabine to malignant cells expressing tumor-associated integrins [11]. In recent years, major pharmaceutical companies and research centers have focused on monoclonal antibody drugs and bi-specific antibody drugs in targeted therapy, as well as CAR-T and immunoassay point inhibitors in immunotherapy [12,13,14]. Immunotherapy achieves anti-tumor therapy by stimulating the body’s immune system. In immunotherapy, mainly immune cell therapy, immune checkpoint inhibitors, tumor vaccines, and immune system regulators, the immune system is used to recognize and regulate the body’s attack on abnormal cell functions [14]. Small peptides, such as the thymic peptide, with their unique advantages in immunotherapy, are also very prominent. The thymic peptide used has been a non-specific adjuvant therapy for various tumors, as it can induce T cell differentiation and development, promoting its proliferation and improving T cell response to the antigen at the same time [15]. This kind of drug enhances the patient’s immunity, with fewer side effects.

The resistance adaptation ability of small peptides in many drug treatments and their fewer toxic side effects indicate their potential application in further developing novel drugs. Due to these medicines’ unique metabolic processes, many new study areas on the pharmaceutical aspects of protein and peptide drugs have recently emerged [16,17]. Many natural and synthetic peptides were characterized in recent decades, and public databases were established, such as APD3 (database of antimicrobial peptides), the Defensins Knowledgebase, the antiviral AVPdb (database of antiviral peptides), the antiparasitic ParaPep, and the CancerPPD (database of anticancer peptides and proteins) [18,19,20,21,22]. Bioactive small peptides are composed of 2–10 amino acids linked by peptide bonds. Studies have found that amino acids such as Trp, Tyr, Met, Gly, Cys, His, and Pro in the peptide chain can significantly improve the bioactivity [23,24]. In other words, compared with traditional chemotherapy drugs, small peptides have several advantages, such as high absorption, small size, well-defined signaling targets, and minimal toxicity. As such, they offer a new promising area of research. Besides, small peptides are just connected by a few peptide linkages, and their small molecular weight makes them easy to be modified and synthesized. Additionally, small peptides can be used as vectors to deliver drugs specifically to every cell and tissue [25]. However, small peptides have some disadvantages, including a short half-life, easy degradation in vivo, poor stability, etc. Many researchers have tried to modify or develop corresponding pseudo-peptide drugs or have combined small peptides with traditional therapy for tumor combination therapy to achieve better results than with a single treatment. The application of several cyclic peptides as noncovalent nuclear targeting molecular transporters of Dox has been reported [26]. Additionally, another work designed new peptides based on the molecular dynamics simulation (MDs) of the matuzumab-EGFR complex in a water environment. These peptides had a higher affinity to the EGFR relative to that previously reported [27]. The peptide modification of anticancer drugs could enhance their activity and selectivity, perhaps even circumventing multi-drug resistance [28]. 

**Figure 1:**
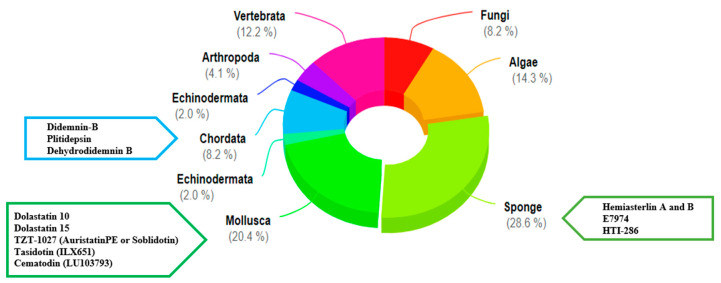
Sources of marine natural products or derivatives that have been approved or entered clinical trials, as well as anticancer small peptides and their derivatives which have entered clinical studies.

**Figure 2:**
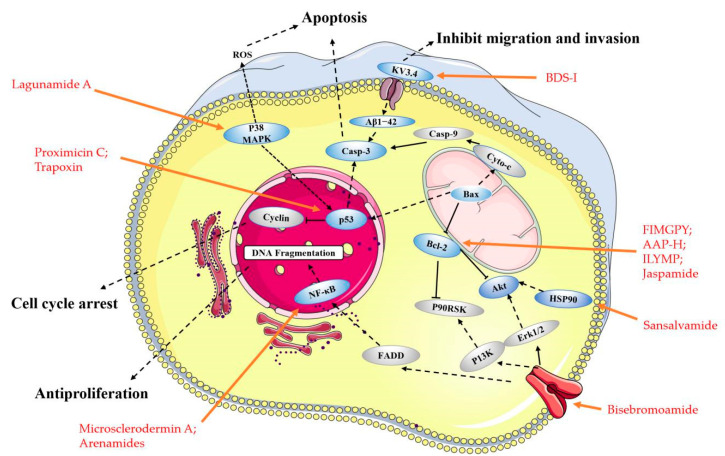
Main molecular mechanism of actions deployed by the anticancer peptides presented in this review. Blue represents the targets mentioned in the paper, and gray represents the proteins involved in the signaling pathway that are not present in this paper, the anticancer peptides mentioned in the article are highlighted in red.

The marine environment is a crucial biological kingdom with the richest source of novel functional proteins and peptides. Additionally, it is gradually becoming a vital field of drug development [29]. As marine organisms live in a special environment that is hypersaline, high-pressure, hypoxia, and hypothermic, and lacks sunlight, these proteins and peptides have strong bioactivities and a specific structure. There are many effects associated with marine peptides, such as antioxidant, antimicrobial, antitumor, antiviral, cardioprotective, immunomodulatory, and tissue regeneration properties [16,30,31,32]. Approximately 49 marine-derived active substances or their derivatives have been approved for the market or entered clinical trials globally [33]. Most of these bioactive molecules are extracted from marine sponges, mollusca, and algae. There are 11 kinds of marine drugs approved by European and American drug authorities, of which, four are listed as anticancer drugs: Cytosar-U, Yondelis, Halaven, and Adcetris. In recent years, more and more studies on marine bioactive peptides have appeared. Many bioactive peptides with anticancer potential have been extracted from various marine organisms. Small peptides from marine sources are gradually gaining attention because of their uniqueness. Compared with high-molecular-weight peptides, low-molecular-weight peptides show greater molecular mobility and diffusivity, contributing to their enhanced interaction with cancer cell components and increasing their anticancer activity [34]. Nowadays, several newly discovered anticancer small peptides and their derivatives thereof from marine organisms have been widely applied to clinical research [35,36,37] (Figure 1). Marine small peptides are molecules that participates in all processes of life activities. They can bear anticancer roles in diversiform aspects in different ways, such as preventing cell migration, induction of apoptosis, disorganization of tubulin structure and inducing cell cycle arrest, and more (Figure 2) [38].

Due to differences in the properties and activities of linear and cyclic peptides, we have generally used these two categories for the review. The first part and the second part of this article introduce the research progress of marine-derived antitumor linear peptides and cyclic peptides, respectively. Finally, we systematically summarized the small peptides and their derivatives that have entered the stage of clinical research.

## 2. Linear Peptides and Derivatives

### 2.1. Animals

In recent years, some anticancer peptides have been discovered from proteolytic products and secondary metabolites of marine animals, and these purified anticancer peptides have cytotoxic, anti-proliferative and protease inhibition effects [39].

The early discovery of the anti-tumor effect of small marine peptides was mostly based on cytotoxicity assessment, and the subsequent mechanism is not clear. Ding Guo-Fang et al. discovered a tripeptide QPK (Table 1) with anticancer activity shows that it inhibited the growth of DU-145 cells (human prostate cancer cells) in a dose-dependent manner, the IC_50_ (half-maximal inhibitory concentration) fell from 9.50 mg/mL at 24 h to 1.00 mg/mL at 48 h [40]. Three novel cytotoxic peptides, AGAPGG, AERQ, and RDTQ (Table 1), were successfully purified and identified from the papain hydrolysate of *Sarcophyton glaucum*. They displayed relatively high cytotoxicity on HeLa cells (human cervical cancer cells), which was 3.3-, 5.8-, and 5.1-fold stronger than that of the anticancer drug 5-FU, respectively. Additionally, their IC_50_ values to inhibit the growth of Hela cells were 8.6, 4.9, and 5.6 mmol/L, respectively [41]. 

**Figure 3:**
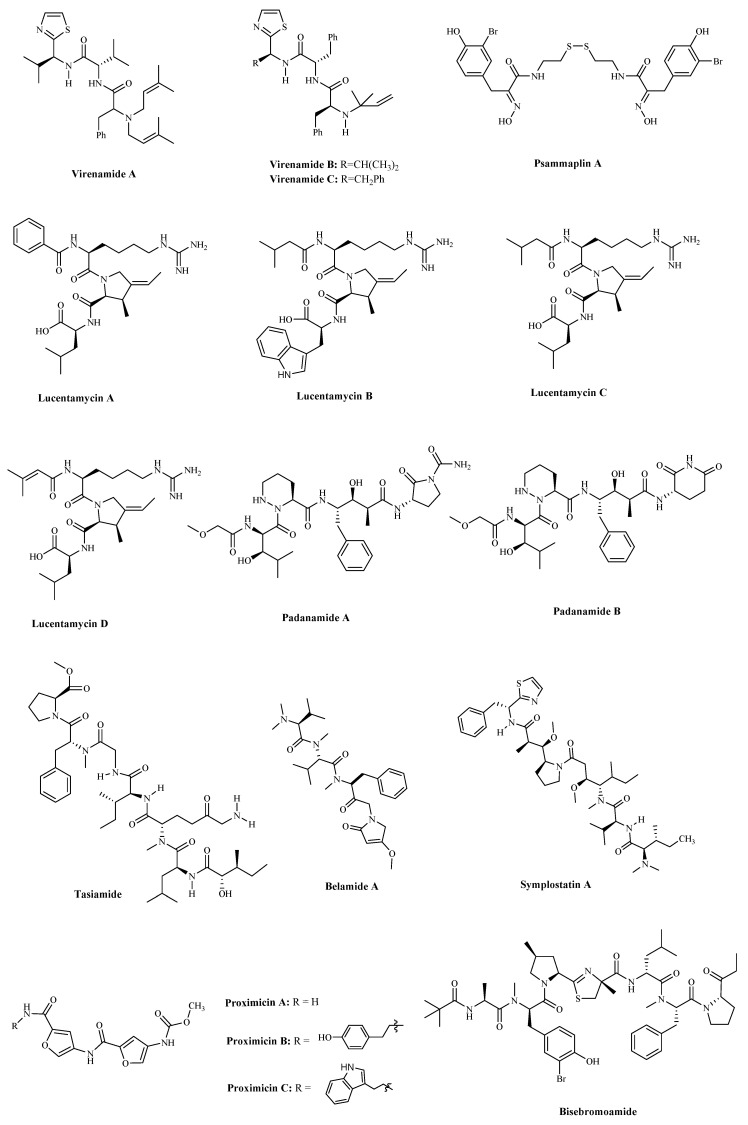
The structures of bioactive marine linear peptides and derivatives with anticancer potential.

Marine natural products are an important source of topological enzyme inhibitors and DNA damaging agents. Virenamides A–C (Table 1 and Figure 3) have been isolated from extracts of the Didemnid ascidian *Diplosoma virens*. It is reported that Virenamide A exhibited topoisomerase II inhibitory activity and had modest cytotoxicity toward a panel of cultured cells: gave an IC_50_ of 2.5 μg/mL against P388 (mouse leukemia cells), and 10 μg/mL against A549 (human non-small cells lung cancer cells), HT-29 (human colon cancer cells), and CV1 (kidney cells) cells. Additionally, Virenamides B and C both had an IC_50_ of 5 μg/mL against P388, A549, HT-29, and CV1 cells [42]. SCAP1 (Table 1) is an anticancer and antioxidative peptide that was shown to initiate cancer cell death by inhibiting cancer cell growth and increasing DNA damage and apoptosis in HT-29 with IC_50_ values of 90.31 to 60.21 μg/mL [43,44]. 

With the deepening of understanding, researchers have discovered that a variety of marine small peptides can induce tumor cell apoptosis to exert anti-tumor effects. The peptide sequence was identified as YALPAH (Table 1), isolated from half-fin *Setipinna taty* anchovy, it has been found to induce PC-3 cells (human prostate cancer cells) apoptosis and inhibit cells proliferation, with an IC_50_ value of 8.1 mg/mL [45]. Additionally, three modified peptides were synthesized ulteriorly. It was revealed that the guanidine portion of arginine (R) forms hydrogen bonds with phosphates, sulfates, and carboxylate salts, which affect the proliferative activity [46]. A tripeptide, BCP-A (Table 1), was isolated from the protein hydrolysate of blood clam (*Tegillarca granosa*) muscle, showing a strong cytotoxicity toward PC-3, DU-145, H-1299 (human lung cancer cells), and HeLa cells with an IC_50_ of 1.99, 2.80, 3.3 and 2.54 mg/mL, respectively. Additionally, it was also displaying a high anti-proliferation activity on the PC-3 cells by inducing apoptosis. In addition, BCP-A has a significant anti-lipid peroxidation effect, which is not conducive to tumor formation [47]. A novel peptide obtained from the sea anemone toxin, BDS-I (Table 1), had been successfully identified as a new inhibitor of the KV3.4 channel subunits. In particular, it had been reported that KV3.4 channels play a crucial role in cancer cell migration [48]. BDS-I blocking KV3.4 currents prevented (the neurotoxic β-amyloid peptide1–42) Aβ1−42-induced caspase-3 activation and apoptotic processes [49,50]. 

Moreover, several small peptides are closely associated with the mitochondrial-mediated apoptosis pathway. The hexapeptide FIMGPY (Table 1), from the skate (*Raja porosa*) cartilage protein hydrolysate, displayed high anti-proliferation activities in HeLa cells with an IC_50_ of 4.81 mg/mL. It also could induce apoptosis by upregulating the Bax/Bcl-2 ratio and caspase-3 activation [51]. The anticancer peptide AAP-H (Table 1) is a pentapeptide from the sea anemone *Anthopleura anjunae* with an amino acid sequence Tyr-Val-Pro-Gly-Pro. It has been shown that AAP-H induces apoptosis by decreasing the mitochondrial membrane potential and increasing Bax/Bcl-2 ratio, cytochrome-C, caspase-3, and caspase-9 [52]. An antiproliferative pentapeptide ILYMP (Table 1), was isolated from the protein hydrolysate of *Cyclina sinensis*. It has been demonstrated that *ILYMP* enhances Bax and cleaved caspase-3/9 expression and the suppression of Bcl-2 expression in DU-145 cells [53].

Apoptosis is closely related to cell cycle arrest. At present, some small peptides discovered cannot only induce cancer cells apoptosis, but also cause cell cycle arrest and ultimately lead to cell death. The sequences of SCH-P9 and SCH-P10 (Table 1), identified as Leu-Pro-Gly-Pro and Asp-Tyr-Val-Pro, were obtained from *Sinonovacula constricta* hydrolysates. The researches illustrated that SCH-P9 and SCH-P10 inhibited the growth of DU-145 cells and PC-3 cells by reducing the number of cells in the G0/G1 phase, thus increasing the number in the sub G1 phase and inducing apoptosis [54]. SIO (Table 1) is another tripeptide found in *sepia* ink. The research found that it significantly inhibited the proliferation of DU-145, PC-3, LNCaP (human prostate cancer cells), A549 and H-1299 cells, in a time and dose-dependent manner by inducing apoptosis and arresting cell at S or G2/M phase [55,56]. The anticancer mechanism is similar to another decapeptide SHP, which is accompanied by the activation of cellular tumor antigen p53 and caspase-3, the upregulation of pro-apoptosis regulator Bax, and the downregulation of anti-apoptosis regulator Bcl-2 [57]. Psammaplin A (PsA) (Table 1 and Figure 3) is a natural product that has been isolated from sponges and has been suggested to be a promising novel HDAC (histone deacetylase) inhibitor. Some researchers found that PsA exhibited antiproliferative effects on cancer cells by the induction of cell cycle arrest and apoptosis. However, the psammaplin class has the disadvantage of physiologic instability [58,59]. Latest research reports that the indole derivatives of Psammaplin are more potent modulators of epigenetic enzymes than the original natural product. Additionally, positional isomers at the bromoindole ring also showed cell cycle block and apoptosis induction [59]. NVP-LAQ824 (Table 1) is a more stable indolic cinnamyl hydroxamate analogue of Psammaplin A, has entered phase I clinical trials in patients with solid tumors or leukemia [60]. A toxicity evaluation in rats identified the hematopoietic and lymphatic systems as the primary target organs, with a reversible dose-dependent reduction in RBC (red blood cell) and WBC (white blood cell) counts and lymphoid atrophy [60].

### 2.2. Fungi and Bacteria

Hundreds of secondary metabolites obtained from marine fungal strains revealed potent pharmacological and biological activities [61]. Lucentamycins A–D (Table 1 and Figure 3), have been isolated from the fermentation broth of a marine-derived actinomycete identified by phylogenetic methods as *Nocardiopsis lucentensis*. Lucentamycins A and B showed significant in vitro cytotoxicity against HCT-116 cells (human colon cancer cells) with IC_50_ values of 0.20 and 11 µM [62]. Two highly modified linear tetrapeptides, Padanamides A and B (Table 1 and Figure 3), were obtained from sediment in the culture of *Streptomyces* sp. It demonstrated that Padanamide B is cytotoxic to Jurkat cells (human leukemia cells) with an IC_50_ value of 30.9 µM [63]. Tasiamide (Table 1 and Figure 3) was predicted to be the best active cyanobacterial compound derived from *Symploca* sp. It has been shown that it was cytotoxic against KB (human nasopharyngeal cancer cells) and LoVo (human colon cancer cells) cells, with IC_50_ values of 0.48 and 3.47 μg/mL, respectively [64]. Cathepsin D (Cath D) has been considered a potential target to treat cancer [65]. Tasiamide’s C-terminal modified derivatives have inhibitory activity against Cath D/Cath E/BACE1, potentially making them highly potent and selective Cath D inhibitors [66]. Belamide A (Table 1 and Figure 3) is a highly methylated linear tetrapeptide, with a structural analogy to the important linear peptides, Dolastatins 10 and 15. It has a moderate intensity of cytotoxicity to HCT-116 cells (IC_50_: 0.74 μM). At a concentration of 20 μM, it destroyed the micro-tubule network in rat aortic smooth muscle A-10 cells and showed the classic tubulin destabilizing mitotic characteristics [67]. Symplostatin *A* (Table 1 and Figure 3), a Dolastatin *10* analogue from the cyanobacterium *Symploca hydnoides*, can cause a loss of interphase micro-tubules, G2/M arrest, and active caspase 3 and initiate the phosphorylation of Bcl-2 [68]. Proximicins A, B, and C (Table 1 and Figure 3) are a family of three novel aminofuran antibiotics isolated from actinomycetes of the genus *Verrucosispora*. It was illustrated that Proximicins could activate cell-cycle regulatory proteins involved in the transition of cells from G1 to S phase and induce apoptotic cell death in L1236 (Hodgkin’s Lymphoma cells) Jurkat 16 (T-cell leukemia cells) cells. Moreover, Proximicin C can induce up-regulation of p53 and p21 in gastric adenocarcinoma cells, and inhibit the U-87 MG (human glioblastoma cells) and MDA-MB-231 (human breast cancer cells) cells proliferation, with IC_50_ values of 12.7 and 11.4 μg/mL, respectively [69]. Bisebromoamide (Table 1 and Figure 3), a cyanobacterial metabolite from a cyanobacterium of the genus, *Lyngbya* sp., was shown to have an antiproliferative activity at nanomolar levels of 40 nM that average a 50% growth inhibition (GI_50_) value across all of the cell lines (a panel of 39 human cancer cell lines (termed JFCR39)) [70]. Additionally, it can also inhibit the Raf/MEK/ERK and PI3K/Akt/mTOR pathways, showing a potent protein kinase inhibitor effect [71]. 

### 2.3. Other Small Peptides

Currently, the number of small linear anti-cancer peptides derived from marine plants is relatively small. The peptide, HVLSRAPR (Table 1), exhibited strong inhibitory activity on HT-29 cells, with an IC_50_ value of 99.88 μg/mL, while it had little inhibitory activity on LO2 cells (normal liver cells) (5.37% at 500 μg/mL). It was also shown to be selectively active on cancer cells, including Hep G2 (human liver cancer cells), MCF-7 (human breast cancer cells), SGC-7901 (human gastric cancer cells), and A549 cells [72]. 

Most of the small peptides are still in the stage of structural optimization and in vitro activity verification. The lack of progress in subsequent research on their mechanisms prevents them from entering clinical development. Additionally, these small peptides merit further investigation as a potential therapeutic agent.

## 3. Cyclic Peptides and Derivatives

Cyclic peptides combine several favorable properties, such as a good binding affinity, target selectivity, and low toxicity, which make them attractive in anticancer drug researches [73]. Besides, most of the small peptides are concentrated in marine animals, and they are found to be secondary metabolites from sponges, sea squirts, cnidaria, and mollusks.

### 3.1. Animals

#### 3.1.1. Metabolites of Ascidians 

**Figure 4:**
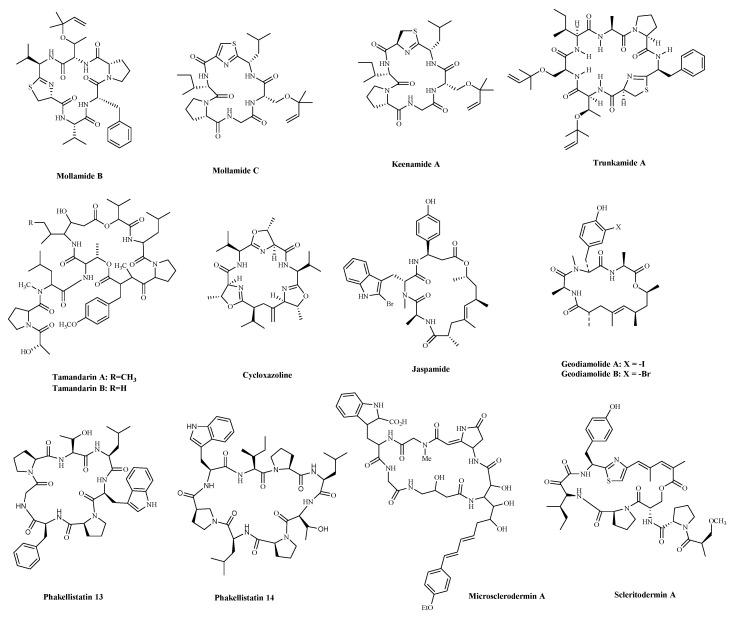
The structures of marine bioactive cyclic peptides from Ascidians and Sponges.

A significant number of compounds with unusual structures and bioactivities have been isolated from various ascidians. The cyclic hexapeptides, Mollamides B and C (Table 2 and Figure 4), were isolated from an Indonesian tunicate *Didemnum mole* in 1994, along with the known peptide, Keenamide A (Table 2 and Figure 4) [74]. Keenamide A and Mollamides B show antiproliferation activity against several cancer cell lines [74]. Keenamide A show cytotoxicity against a range of cell lines, with IC_50_ values of 2.5 μg/mL toward P388, A549 and MEL-20, and 5.0 μg/mL against HT-29 cells [75]. Mollamides B inhibited the proliferation of H460 (human non-small cell lung cancer cells) and MCF-7 and SF-268 cells, but the GI_50_ values are all greater than 100 μM [74]. Subsequently, the cyclic depsipeptides Trunkamide A (Table 2 and Figure 4) was isolated from the colonial ascidian *Lissoclinum* sp., which was collected by Bowden and co-workers in 1996, and its structure was similar to Didemnin B. It has been found to have a cytotoxic effect on several human cancer cell lines, such as HeLa, AGS (human gastric cancer adenocytes cells), and DLD-1 (human colonic adenocarcinoma cells) cells [76]. Tamandarins A and B (Table 2 and Figure 4), were isolated from the unidentified species of didemnid ascidian in 2000 and determined to be cyclic depsipeptides closely related to Didemnins. They were found to possess a very similar structure and biological activity to that of the Didemnin B. They were evaluated against various human cancer cell lines and slightly more potent than Didemnin B [77]. Cycloxazoline (Table 2 and Figure 4) is an asymmetrical cyclic hexapeptide isolated from species of didemnid ascidians in 1992. It can delay S-phase cells entering the G2/M phase and induce apoptosis in HL-60 human leukemia cells [78]. These peptides are structurally similar to Didemnin B, but it’s unclear whether they have a better effect or mechanism on tumor cells.

#### 3.1.2. Metabolites of Sponges

Sponges are an excellent source of bioactive metabolites with novel chemical architectures. They produce a diverse array of highly modified peptides, especially cyclic peptides with nonproteinogenic amino acids and polyketide-derived moieties. The depsipeptides isolated from sponges or associated organisms are usually described as cytotoxic substances, such as Jaspamide (Jasplakinolide) [79], Geodiamolides [80], Phakellistatin [81], Microsclerodermin A [82] and Scleritodermin A [83]. However, some of those researched have an anticancer mechanism for a specific cancer cell. Jaspamide (jasplakinolide, NSC-613009, Table 2 and Figure 4), isolated from the sponge, *Jaspis Johnstoni* in 1986, has been considered a classical actin stabilizer [84]. Jaspamide-induced apoptosis is associated with caspase-3 activation, and increased Bax level, and decreased Bcl-2 protein expression. It exhibits antitumor activity in multiple in vitro tumor models for prostate and breast carcinomas and acute myeloid leukemia [85,86]. Geodiamolides A, B (Table 2 and Figure 4) were isolated from the sponge *Geodia* sp. in 1987 [87,88]. In a way similar to other depsipeptides (Jaspamide and Dolastatins), it keeps the normal microtubule organization and regulates actin cytoskeleton, migration, and invasion of breast cancer cells [89,90]. Phakellistatins, a class of cycloheptapeptides, isolated from *Phalkellia* sp., are potent anti-proliferative agents against the leukemia cell line. Phakellistatin 14 (Table 2 and Figure 4) showed cancer cell growth inhibitory activity (GI_50_: 5 μg/mL) against P388 cells, and Phakellistatins 13 is reported to be cytotoxic at an effective dose of 10 ng/mL (GI_50_) against BEL-7404 (human liver cancer cells) cells [91,92]. Due to its good antitumor activity in vitro and in vivo, many researchers have modified this structure in the hope of synthesizing potential Marine drugs with a better anti-cancer effect [93]. The Microsclerodermins are cyclic hexapeptides isolated from a deep-water sponge of the genus *Microscleroderma* in 1994. Microsclerodermin A (Table 2 and Figure 4) has been demonstrated to inhibit NF-κB and induce apoptosis in the AsPC-1, BxPC-3, and PANC-1 pancreatic cancer cell lines, its IC_50_ values were 2.3, 0.8, 4.3, and 4.0 μM against the four cells, respectively [82]. Additionally, recent research has discovered the congeners of the Microsclerodermins, Microsclerodermins N and O. They exhibit cytotoxic activity against HeLa cells with IC_50_ values of 0.77 mM and 0.81 mM, respectively [94]. Scleritodermin A (Table 2 and Figure 4) was isolated from the lithistid sponge *Scleritoderma nodosum* in 2004. Scleritodermin A has significant in vitro cytotoxicity against a panel of human tumor cell lines and acts through tubulin polymerization inhibition and the resulting disruption of microtubules [83]. Subsequent studies have led to discovering several novel cyclopeptides, but their antitumor activity is relatively weak, and the mechanism of action is unclear [95]. These peptides have unique structures as compared with those from other sources. This attribute makes sponge- and tunicate-derived peptides highly attractive as potential drug and molecular probes.

### 3.2. Fungi

**Figure 5:**
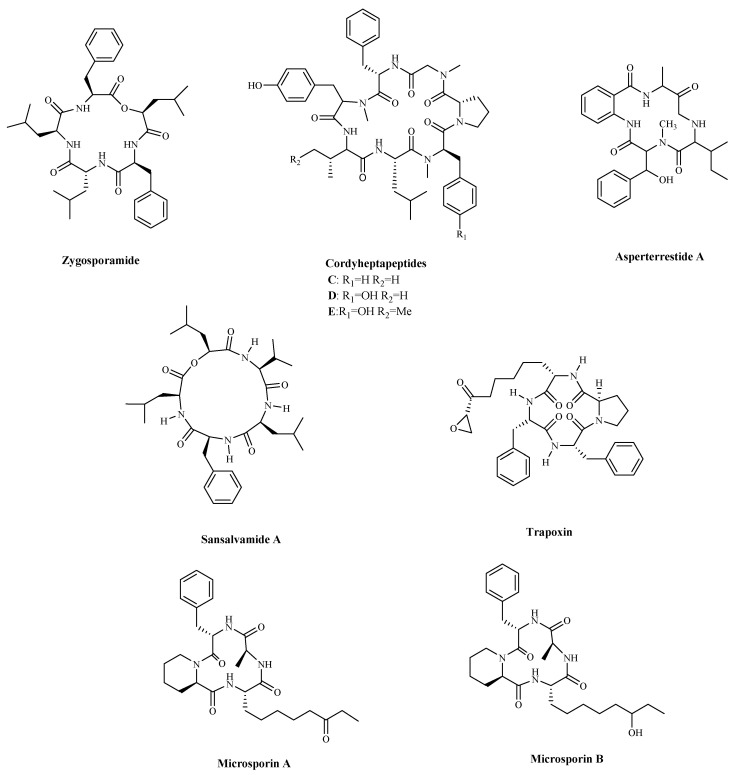
The structures of marine bioactive cyclic peptides from Fungi.

Zygosporamide (Table 3 and Figure 5), a new cyclic pentadepsipeptide, was isolated from the seawater-based fermentation broth of a fungus identified as *Zygosporium masonii.* Zygosporamide showed a significant cytotoxicity in the NCI’s 60 cell line panel (GI_50_ = 9.1 μM), with a highly enhanced selectivity against the central nervous system cancer cells SF-268 (GI_50_ = 6.5 nM), and the renal cancer cells RXF 393 (GI_50_ = 5.0 nM) [96]. Three new cycloheptapeptides, Cordyheptapeptides C–E (Table 3 and Figure 5), were isolated from the fungus fermentation extract, *Acremonium persicinum* SCSIO 115. The compounds displayed cytotoxicity against SF-268 (Human neuro cancer cell), MCF-7, and NCI-H460 tumor cell lines, with IC_50_ values ranging from 2.5 to 12.1 μM [97]. A new cytotoxic and antiviral cyclic tetrapeptide, Asperterrestide A (Table 3 and Figure 5), showed cytotoxicity against U937 and MOLT4 (human acute T lymphoblastic leukaemia cells) cells and inhibitory effects on influenza virus strains H1N1 and H3N2 [98]. Unfortunately, although these compounds have good anti-cancer activity in vitro, their specific anti-cancer mechanism is not clear.

Sansalvamide A (Table 3 and Figure 5) is a cyclic depsipeptide produced by the fungi *Microsporum* cf. *gypseum*. Since ring-opening enzymes easily inactivate natural products, many analogues have been synthesized and modified to provide better stability, and 86 analogues have been reported synthesized [99]. Recently researchers have shown that Sansalvamide A and Sansalvamide analogues inhibit cell growth and proliferation, and induce cell apoptosis by regulating the expression of HSP90 [99,100]. New research has shown that HSP90A strengthens AKT activation through TCLlA-stabilization, promoting multi-aggressive properties in tumor cells [101].

Trapoxin (Table 3 and Figure 5), is a known potent irreversible inhibitor of histone deacetylase. The report indicated that the inhibitory warhead is the α, β-epoxyketone side-chain of (2S,9S)-2-amino-8-oxo-9,10-epoxydecanoic acid (l-Aoe) [102,103]. Some finding displayed that the primary target molecule of the agent in vivo is the histone deacetylase itself, and it also induced growth inhibition in several cell lines regardless of their p53 status [104,105]. Other cyclic peptides, Microsporins A and B (Table 3 and Figure 5) were isolated from culture extracts of the fungus, *Microsporum* cf. *gypseum*. Microsporins A and B are potent inhibitors of histone deacetylase, with IC_50_ values equal to 0.6 mg/mL and 8.5 mg/mL against HCT-116 cells. The results of the HDAC enzyme activity inhibition experiment showed that Microsporins A had a greater inhibitory effect against HDAC_S_ and HDAC8 than SAHA, with IC_50_ values of 0.14 and 0.55 µM, respectively [106].

### 3.3. Bacteria

**Figure 6:**
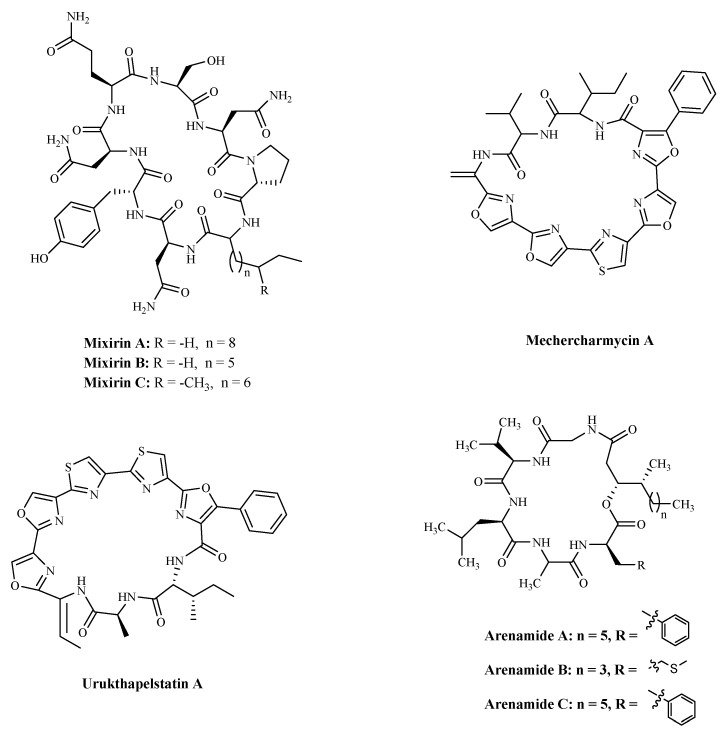
The structures of marine bioactive cyclic peptides from Bacteria.

Bacterial proteins and peptides are a class of promising bioactive compounds and potential anticancer drugs [107]. Mixirins are cyclic acyl-peptides derived from the bacterium *Bacillus* species. Mixirins A, B and C (Table 4 and Figure 6) blocked the growth of the HCT-116 with an IC_50_ value at the level of 0.65, 1.6, and 1.26 µM, respectively [108]. A new cytotoxic substance named Mechercharmycin A (Table 4 and Figure 6) was isolated from *Thermoactinomyces* sp. YM3-251 showed relatively strong antitumor activity against A549 and Jurkat cells with an IC_50_ value of 40 nM and 46 nM, respectively [109]. Urukthapelstatin A (Table 4 and Figure 6), a novel cyclic peptide, was isolated from the cultured mycelia of Thermoactinomycetaceae bacterium *Mechercharimyces asporophorigenens* YM11-542. Research showed that Urukthapelstatin A inhibited human growth lung cancer A549 cells with an IC_50_ value of 12 nM. Recently analogues of Urukthapelstatin A were synthesized. Cytotoxicity data showed the phenyl ring attached to the eastern oxazole and the rigid, lipophilic tripeptide section are critical structural features of the bio-activity [110]. Three new cyclohexadepsipeptides, Arenamides A–C (Table 3 and Figure 6), were isolated from the fermentation broth of a bacterial strain, identified as *Salinispora arenicola*. Arenamide A and B’s effect on NF-κB activity was studied with stably transfected 293/NF-κB-Luc human embryonic kidney cells, induced by treatment with tumor necrosis factor (TNF). Arenamides A and B blocked TNF-induced activation in a dose- and time-dependent manner with IC_50_ values of 3.7 and 1.7 μM, respectively [111].

#### Cyanobacterial Metabolites

The potential of marine cyanobacteria, as anticancer agents, displays selective cytotoxicity in tumor cell lines. The mechanism of action of these compounds is not the same, including induction of apoptosis, inhibition of protease, inhibition of HDAC and other mechanisms.

**Figure 7:**
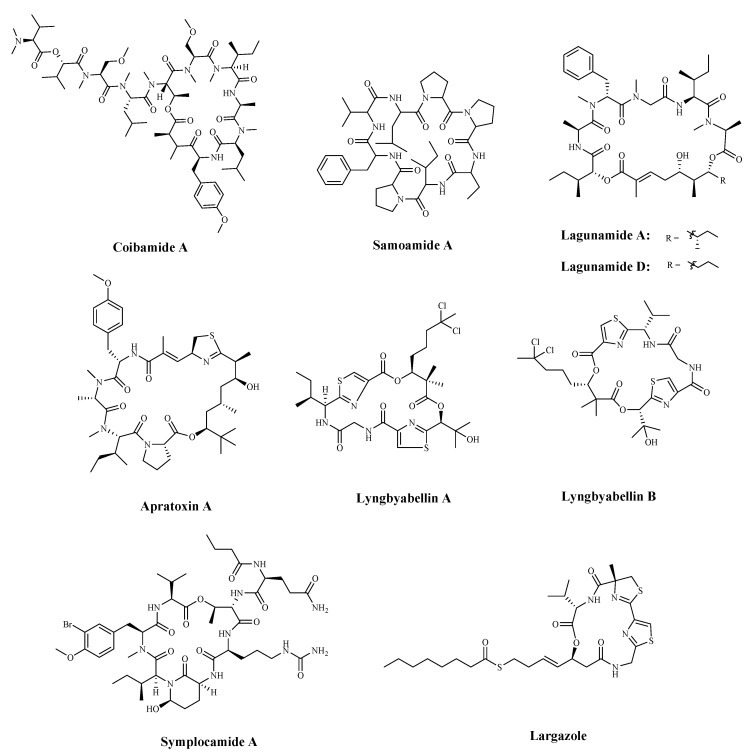
The structures of marine bioactive cyclic peptides from cyanobacteria.

Coibamide A (Table 5 and Figure 7) is a new, potent anti-proliferative depsipeptide, which was isolated from the *Leptolyngbya* cyanobacterium. It displayed a potent cytotoxicity against NCI-H460 lung cancer cells and mouse neuro-2a cells (IC_50_ < 23 nM) [112]. Samoamide A (Table 5 and Figure 7) is a cyclic peptide extracted from *Symploca* sp., collected in American Samoan. It has shown a good cytotoxicity in vitro activity tests, with an IC_50_ value of < 10 μM against colorectal cancer cells and between 1.1 and 4.5 μM against non-small lung cancer cells, breast cancer cells, and others [113,114]. Lagunamide A (Table 5 and Figure 7), a cytotoxic cyclodepsipeptide isolated from cyanobacterium, *Lyngbya majuscule*, has been found to induce caspase-mediated mitochondrial apoptosis, accompanied by the dissipation of mitochondrial membrane potential (Δφm) and the overproduction of reactive oxygen species (ROS) [115]. Furthermore, Lagunamide D (Table 5 and Figure 7) displayed potent activity in triggering apoptosis in a dose- and time-dependent manner [116]. Its structure is closely related to a series of marine-originated compounds from cyanobacteria, including Aurilides [117], Odoamide [118], Palau’amide [119]. The cyanobacterial metabolite Apratoxins (Table 5 and Figure 7) are a family of potent anticancer and antiangiogenic agents. Apratoxins A (Table 5 and Figure 7) down-regulated receptors and growth factor ligands for cancer cells that rely on autocrine loops [120]. Recently, a novel Apratoxin analogue, Apratoxin S10 (Apra S10), has been reported. It inhibited pancreatic cancer cell secretion and reduced the factors secreted by other cell types active within the tumor microenvironment [121]. Lyngyabellins A and B (Table 5 and Figure 7) display anti-proliferative activities in various cell types. Lyngbyabellin A has been demonstrated to have potent cytotoxic activities against human cancer cell lines. It induces apoptosis through the disruption of cellular microfilament network cytokinesis [122]. 

Cyanobacterial serine protease inhibitors are the most predominant secondary metabolites isolated from cyanobacteria. Some new protease inhibitor compounds were found in cyanobacteria, such as Symplocamide A (Table 5 and Figure 7) [123]. The presence of several unusual structural features in Symplocamide A provides new insights into the pharmacophore model for protease selectivity in this drug class. It has been found that it inhibits serine proteases, with a 200-fold greater inhibition of chymotrypsin over trypsin, which may underlie the potent cytotoxicity to H-460 lung cancer cells (IC_50_ = 40 nM), as well as neuro-2a neuroblastoma cells (IC_50_ = 29 nM) [123]. 

Largazole (Table 5 and Figure 7), isolated from a cyanobacterium of the *Symploca* genus, was shown to be effective in inhibiting tumor growth and induced apoptosis in a tumor. This selectivity is attributed to its very potent class I HDAC inhibitory activity. As it has various biological activities, most Largazole analogues have been synthesized to improve its activity [124,125].

## 4. Marine-Derived Small Peptides in Clinical Trials

Some marine small peptides have entered the clinical stage. However, due to their shortcomings such as low activity and large side effects, some have been terminated. What is gratifying is that a large number of researchers have synthesized a series of derivatives by optimizing their structures, and some of them are currently in the clinical stage. In this review, we collected the current clinical information and the latest clinical progress of anti-tumor small peptides through clinicaltrials.gov, PubMed, and Scopus, etc. 

### 4.1. Linear Peptides

**Figure 8:**
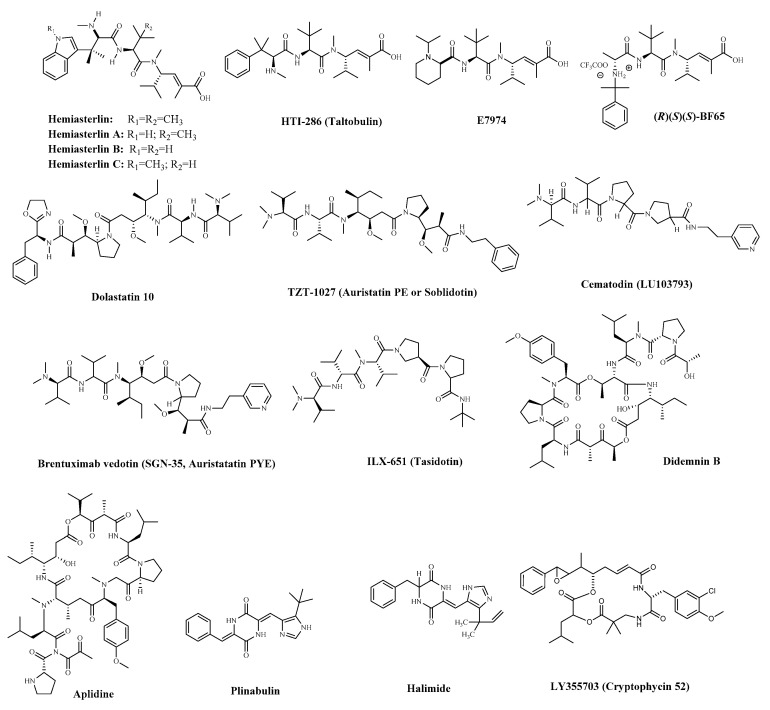
Marine-derived clinical small peptides and some of their derivatives.

The Hemiasterlins (Figure 8) are a family of potent cytotoxic peptides isolated from sponges [126]. Like other peptide molecules with a diversity of structure, Hemiasterlins bind to the “Vinca-peptide site” in tubulin, disrupts the normal microtubule dynamics, and, at stoichiometric amounts, depolymerizes microtubules [127,128]. They progressed into Phase II, but the trials were discontinued due to significant bone marrow toxicity and neuropathy. The structural motif of the Hemiasterlins would appear to be readily amenable to the rapid generation of analogues by combinatorial chemistry. Two novel synthetic analogues of Hemiasterlin are known as HTI-286 (Taltobulin, Table S1 and Figure 8) and E7974 (Table S1 and Figure 8). They have been reported to have potent activity against tumor cells with a potentially high therapeutic index [129,130,131,132,133,134,135,136]. A synthetic analogue of Hemiasterlin, HTI-286, has significantly improved the drug resistance [130]. Some researchers found the N-isopropyl-d-pipecolic acid derivative E7974, which retains the potent in vitro antitumor activity of Hemiasterlin and induces a long-lasting mitotic blockade that ultimately triggers apoptosis [129]. The development of Hemiasterlin derivatives *BF65* synergized with a colchicine site microtubule inhibitor stilbene 5c both in vitro and in vivo, which may provide a potential drug combination in future clinical application [132]. It was reported that the stereospecific diastereomer (R)(S)(S)-BF65 (Figure 8) could synergize with an allosteric Akt inhibitor MK-2206 to suppress the growth of SKOV3 ovarian cancer cells [130]. Hemiasterlin derivatives HTI-286 and E7974 are poor substrates for P-glycoprotein and can circumvent drug resistance. All of them have entered the clinical stage (clinical information is shown in Table S1). However, it remains to be determined whether Hemiasterlins and derivatives have a better therapeutic profile for clinical development.

Dolastatins are a series of cytotoxic peptides that were originally isolated from the sea hare *Dolabella Auricularia* as early as 1972 and recently obtained from a cyanobacterium. However, the most potent constituent Dolastatin 10 (Table S1 and Figure 8) was not until 1987 that it was isolated and characterized. Dolastatin 10 was a linear pentapeptide with four distinctive amino acids and exhibited potent inhibitory activity against a battery of human cancer cell lines [133]. It has been shown that Dolastatin 10 inhibits the binding of vincristine alkaloids to tubulin in a competitive inhibitory manner and binds adjacent to the exchangeable GTP site on the β-tubulin [134,135]. Dolastatin 10 progressed through to Phase II trials as a single agent. However, it did not demonstrate significant antitumor activity in a Phase II trial, together with problems of complex chemical synthesis with low yields and poor water solubility. Therefore, a large number of derivatives have been synthesized to optimize the structure and enhance the antitumor activity. Specific clinical information is shown in Table S1. Due to various harmful side effects with these series of cytotoxic peptides, they have been synthesized and modified largely, some of the derivatives are already in clinical trials, such as TZT-1027 (Auristatin PE or Soblidotin, Table S1 and Figure 8) [136,137,138,139]. Cematodin (LU103793, Figure 8) [140] and Glembatumumab vedotin (CDX-011, Table S1) [141]. Their clinical information is shown in Table S1. TZT-1027 is an anti-microtubule agent, which has been demonstrated to have a potent anti-vascular effect in the advanced stage of vascular-rich SBC-3/VEGF tumors and is currently in Phase II clinical trials [138]. However, significant side effects were observed in clinical trials at dose levels that were insufficient to attain clinical efficacy [142]. Auristatin PYE (SGN 35, Table S1 and Figure 8) is a novel synthetic derivative of Dolastatin 10 with a structural modification of phenol to a pyridine from Auristatin PE. It was reported that Auristatin PYE was less potent in vitro than Dolastatin 10, but it was significantly more effective (*p* < 0.01) in vivo against both human colon adenocarcinoma cell lines [143]. Owing to the drug resistance of tumor cells, Auristatin PYE could be exploited in combination therapy to improve the efficacy of the standard agent [144].

Similar to the drug Dolastatin 10, Dolastatin 15 is an anti-neoplastic pseudopeptide that represses tubulin-dependent GTP hydrolysis to tubulin. The derivative of Dolastatin 15 is known as Cematodin or LU-103793 (Figure 8). It has been illustrated that Cemadotin inhibits cell proliferation by blocking mitosis at the G2/M phase in lymphoma cell lines and was entered into Phase I clinical trials for treatment of breast cancer. It progressed into Phase II, but trials appear to have been discontinued to strong side-effects [140]. A new generation of Dolastatins represented by Tasidotin (ILX-651, Table S1 and Figure 8) and its major metabolite Tasidotin C-carboxylate, restrain dynamic instability at the plus ends of purified microtubules in vitro at concentrations that are 10 to 20 times lower than those which inhibit microtubule polymerization, and the metabolite is considerably more potent than that of the parent compound [145]. In Phase I clinical trials, Tasidotin was found to have several advantages over other Dolastatins for treating patients with advanced solid tumors, including reduced toxicity and the absence of long-term accumulation [146,147,148].

While it was designed as a single-agent drug from a Dolastatin analogue, ADCs selectively transport drugs to target sites, increasing drug activity while reducing side effects through selectivity. Monomethyl auristatin E (MMAE), as an ADC, has been utilized to increase antitumor activity. For example, Glembatumumab vedotin (CDX-011; formerly CR011-vcMMAE, Table S1) is an antibody–drug conjugate consisting of CR011, a fully human IgG2monoclonal antibody against gpNMB, conjugated via a valine-citrulline link to the potent microtubule inhibitor, MMAE [149]. Therefore, the FDA granted Fast Track designation to CDX-011 for the treatment of advanced, refractory, or resistant GPNMB-expressing breast cancer and was entered into phase II study to evaluate the overall response rate and safety of GV, glycoprotein NMB (GPNMB) expression, and survival in patients with metastatic uveal melanoma [141,150]. Similarly, Brentuximab vedotin (SGN-35, Table S1 and Figure 8) is an ADC comprising an anti-CD30 antibody conjugated by a protease-cleavable linker to the potent anti-microtubule agent, MMAE [149,151]. It gained the United States Food and Drug Administration (FDA) approval in 2011 for the treatment of Hodgkin lymphoma and systemic anapla sms known to stic large cell lymphoma [36].

### 4.2. Cyclic Peptides

The peptide metabolite Didemnin B (Table S1 and Figure 8) is a classic type of anticancer cyclic peptides that was the first marine natural product to enter phase I and II clinical trials. It has a number of non-proteinogenic amino acids and contains l-2-hy-droxyisovalerylproprionic acid and l-isostatin within its ring structure. It was shown to induce the death of various transformed cells with apoptotic morphology and DNA fragmentation within the cytosol and the generation of DNA ladders. It also behaved as a potent inhibitor of protein synthesis. However, NCI trials were terminated in 1990 due to toxicity issues [152,153]. Aplidine (Plitidepsin and Dehydrodidemnin B, Table S1 and Figure 8) is a new marine anticancer depsipeptide with a chemical structure very similar to that of Didemnin B, has an oxidized pyruvate instead of lactate [154,155]. It has nearly entered a phase II clinical trial with metastatic, relapsed/refractory Dedifferentiated Liposarcoma, but it also can cause common adverse events like nausea, vomiting, and transient transaminitis [156]. It was recently used to evaluate the safety profile of Aplidine in Patients With COVID-19 (APLICOV-PC) [157] (shown in Table S1). Plinabulin (NPI-2358, Table S1 and Figure 8), a potent microtubule-targeting agent derived from the natural diketopiperazine, ’phenylahistin’ with a colchicine-like tubulin depolymerization activity, is an anticancer agent undergoing Phase II clinical trials in four countries, including the United States [158,159]. Another cyclic dipeptide, Halimide (Table S1 and Figure 8), can inhibit spindle formation in cells, thus reducing or inhibiting the ability of the cells to proceed through mitosis, arresting the cells in a premeiotic stage, and inhibiting tubulin polymerization [160]. Cryptophycin (Crp) is a cyanobacterial depsipeptide, which is a new potent cytotoxic anti-microtubule agent. The treatment of cells with Cryptophycin can rapidly cause morphological changes and DNA strand breakage [161,162]. LY355703 (Cryptophytic 52) (Table S1 and Figure 8) is a synthetic derivative of the cryptophycins, it is a cytotoxic agent that induce mitotic arrest by binding at the microtubule vinca binding domain. At present, it has entered phase I clinical treatment [163].

## 5. Conclusions

Due to its nonspecific selectivity and multidrug resistance (MDR), chemotherapy still has numerous cancer treatment challenges [164]. Nowadays, many scientists focused on using delivery systems with nanoscale therapeutics to improve the accuracy and precision of drugs [165]. The chemical diversity and structural complexity of marine natural products show that they may be an unexploited source of structures for use as biological probes or in drug discovery and development. It is reported that some anticancer peptides have high efficacy and selectivity in cancer treatment [166]. Additionally, they are expected to become the ideal drug carriers due to due to their superior targeting ability and low immunogenicity [167]. Among anticancer peptides, cyclic peptides have significant structural advantages. They display a large surface area, which provides a high affinity and selectivity for protein targets. Furthermore, cyclic peptides have little to no toxicity due to their benign amino acid make-up. Thus, they are simple to modify, handle, and characterize, which are all essential properties for therapeutics [168].

Personalized cancer vaccines targeting patient-specific neoantigens constitute a novel model of cancer treatment [169]. However, the neoantigen physicochemical variability is a primary problem associated with the optimal format for combatting cancer by manufacturing personalized cancer vaccines [170]. Anticancer peptide-conjugate vaccine modalities constitute a new potential cancer treatment [171].

Marine small peptides have the unique advantage of less adverse effects than other anti-cancer drugs, and are considered to have better therapeutic effects. At present, a variety of small peptide drugs have entered clinical research (Table S1). Understanding the molecular mechanisms of action about some new bioactive small peptides obtained from natural sources on specific cellular targets contributes to the development of peptides as promising lead drug candidates. This article reviewed small peptides of marine origin with antitumor activity and their action mechanism in recent years. Hopefully, it will serve as a reference for the development of novel marine antitumor agents.

## Figures and Tables

**Table 1:** Marine sources of bioactive linear peptides with anticancer potential.

Compound	Source	Mechanism	Cell Lines	IC_50_/(GI_50_) ^b^	Reference
QPK	*Sepia* ink	Cytotoxicity ^a^	DU-145	9.50 mg/mL (24 h);	[40]
				1.00 mg/mL (48 h)	
AGAPGG;	*Sarcophyton glaucum*	Cytotoxicity ^a^	HeLa	8.6 mmol/L;	[41]
AERQ;				4.9 mmol/L;	
RDTQ				5.6 mmol/L	
Virenamides A;	The Didemnid ascidian *Diplosoma virens*	Inhibiting the Topoisomerase II	P388;	2.5 µg/mL	[42]
			A549;	10 µg/mL	
			HT-29;	10 µg/mL	
			CV1	10 µg/mL	
Virenamides B	The Didemnid ascidian *Diplosoma virens*	Inhibiting the Topoisomerase II	P388;	5 µg/mL	
			A549;	5 µg/mL	
			HT-29;	5 µg/mL	
			CV1	5 µg/mL	
Virenamides C	The Didemnid ascidian *Diplosoma virens*	Inhibiting the Topoisomerase II	P388;	5 µg/mL	
			A549;	5 µg/mL	
			HT-29;	5 µg/mL	
			CV1	5 µg/mL	
SCAP1; (Leu-Ala-Asn-Ala-Lys)	Oyster *(Saccostrea cucullata)*	Enhancing oxidative DNA damage; Inducing apoptosis	HT-29	90.31 mg/mL (24 h);	[43,44]
				70.87 mg/mL (48 h);	
				60.21 mg/mL (72 h)	
YALPAH	Half-fin anchovy (*Setipinna taty*)	Inducing apoptosis	PC‑3	8.1 mg/mL	[45,46]
BCP-A (Trp-Pro-Pro)	Blood clam *(Tegillarca granosa)* muscle	Inducing apoptosis and inhibiting lipid peroxidation	PC-3;	1.99 mg/mL;	[47]
			DU-145;	2.80 mg/mL;	
			H-1299;	3.3 mg/mL;	
			HeLa	2.54 mg/mL	
BDS-I; (Ala-Ala-Pro-Ala-Phe-Ala-Ser-Gly)	The sea anemone toxin	Blocking KV3.4 currents prevented (the neurotoxic β-amyloid peptide1-42) Aβ1−42-induced caspase-3 activation and apoptotic processes	PC-12	75 nM	[49,50]
FIMGPY	The skate (*R. porosa*) cartilage protein hydrolysate	Inducing apoptosis by upregulating the Bax/Bcl-2 ratio and caspase-3 activation	HeLa	4.81 mg/mL	[51]
AAP-H; (Tyr-Val-Pro-Gly-Pro)	The sea anemone *Anthopleura anjunae*	Inducing apoptosis, decreasing the mitochondrial membrane potential, and increasing Bax/Bcl-2 ratio, cytochrome-C, caspase-3, and caspase-9	DU-145	9.605 mM (24 h);	[52]
				7.910 mM (48 h);	
				2.298 mM (72 h)	
ILYMP	*Cyclina sinensis*	Enhancing expression of Bax, cleaved caspase-3/9 as well as suppression of Bcl-2 expression	DU-145	11.25 mM	[53]
SCH-P9 (Leu-Pro-Gly-Pro)	*Sinonovacula constricta* hydrolysates	Inducing apoptosis and sub-G1 phase cell cycle arrest	DU‑145;	1.21 mg/mL (24 h);	[54]
			PC‑3	1.09 mg/mL (24 h)	
SCH-P10 (Asp-Tyr-Val-Pro)			DU‑145;	1.41 mg/mL (24 h);	
			PC‑3	0.91 mg/mL (24 h)	
SIO	*Sepia* ink	Inducing apoptosis, and S and G2/M phase cell cycle arrest	DU-145;	<5 mg/mL	[55,56]
			PC-3;	<5 mg/mL	
			LNCaP	<10 mg/mL	
Psammaplin A (PsA)	The two Sponges, *Jaspis* sp.and *Poecillastra wondoensis*.	Inducing S or S-G2/M phase cell cycle arrest; Inhibting HDAC	P388; HCT-116; A549	(40 nM)	[58,59]
NVP-LAQ824	*Psammaplysilla* sp.	Inducing S or S-G2/M phase cell cycle arrest; Inhibting HDAC	H-1299	150 nM	[60]
			HCT-116	10 nM	
Lucentamycins A;	The fermentation broth of a marine-derived actinomycete	Cytotoxicity ^a^	HCT-116	0.20 µM;	[62]
Lucentamycins B				11 µM	
Padanamides A and B	Sediment in the culture of *Streptomyces* sp.	Cytotoxicity ^a^	Jurkat	30.9 µM	[63]
Tasiamide	Cyanobacterial compound derived from *Symploca* sp.	Inhibiting the expression of Cath D	KB;	0.48 μg/mL;	[64,66]
			LoVo	3.47 μg/mL	
Belamide A	Cyanobacterium	Tubulin polymerization inhibition	HCT-116;	0.74 μM;	[67]
			A-10	20 μM	
Symplostatin A	Cyanobacterium	Microtubule assembly Inhibiting cell cycle arrest	MDA-MB-435	0.15 μM	[68]
			SK-OV-3;	0.09 μM	
			NCI/ADR;	2.90 μM	
			NCI/ADR with Verapamil;	0.09 μM	
			A-10;	1.8 μM	
			HUVEC	0.16 μM	
Proximicins C	Actinomycetes of the genus *Verrucosispora,*	Inducing Cell cycle G1 to S phase arrest and inducing apoptotic cell death	U-87 MG;	12.7 μg/mL	[69]
			MDA-MD-231	11.4 μg/mL	
Bisebromoamide	Cyanobacterium of the genus *Lyngbya* sp.	Inhibiting both the Raf/MEK/ERK and PI3K/Akt/mTOR pathways	JFCR39	(40 nM)	[70,71]
HVLSRAPR	*Spirulina platensis*	Cytotoxicity ^a^	HT-29;	99.88 µg/mL	[72]

Notes: ^a^ Mechanism is yet to be investigated; ^b^ If there are parentheses around the value, it means the GI_50_ value is displayed.

**Table 2:** Marine bioactive cyclic peptides from Ascidians and Sponges.

Compound	Source	Mechanism	Cell Lines	IC_50_/(GI_50_) ^b^	Reference
Mollamide B	Tunicate Didemnum	Cytotoxicity ^a^	H460;	(>100 µM)	[74]
			MCF-7;		
			SF-268		
Keenamide A	Tunicate Didemnum.	Cytotoxicity ^a^	P-388;	2.5 µg/mL;	[75]
			A-549;	2.5 µg/mL;	
			MEL-20;	2.5 µg/mL;	
			HT-29	5.0 µg/mL	
Trunkamide A	Didemnid ascidians	Cytotoxicity ^a^	DU-145;	7.08 nM;	[76]
			IGROV;	7.31 nM;	
			SK-BR-3;	5.44 nM;	
			Hela	3.90 nM	
Tamandarin A	Didemnid ascidians	Cytotoxicity ^a^	NCI-60	1.4 µM (2.3 µM)	[77]
Tamandarin B			NCI-60	1.4 µM (2.3 µM)	
Cycloxazoline	Didemnid ascidians	Cell cycle G2/M arrest, Induction of apoptosis	MRC5CV1; T24	0.5 μg/mL	[77]
Jaspamide (Jasplakinolide, NSC-613009)	Sponge *Jaspis johnstoni*	Induced apoptosis is associated with caspase-3 activation, increased Bax level, and decreased Bcl-2 protein expression	T24; MCF-7; 15NCI/ADR; A-10	60 to 150 µg /mL	[85,86]
Geodiamolide A	Sponge *Geodia corticostylifera*	Induction of apoptosis; Tubulin polymerization inhibition	T47D;	18.82 nM;	[89]
			MCF7	17.83 nM;	
Geodiamolides B			T47D;	113.90 nM;	
			MCF7	9.82 nM;	
Phakellistatin 13	Sponge *Phakellia* sp.	Induction of both intrinsic and extrinsic apoptosis	BEL-7404	(10 ng/mL)	[91,92]
Phakellistatin 14			P388	(5 µg/ mL)	
Microsclerodermin A	Sponge of the genus *Amphibleptula*	Inhibit NFκB, Induction of apoptosis;	AsPC-1;	2.3 μM;	[82]
			BxPC-3;	0.8 μM;	
			MIA PaCa-2;	4.3 μM;	
			PANC-1;	4.0 μM	
Scleritodermin A	Sponge *Scleritoderma nodosum*	Tubulin polymerization inhibition	HCT-116;	1.9 µM;	[83]
			A2780;	0.940 µM;	
			SKBR3	0.670 µM	

Notes: ^a^ Mechanism is yet to be investigated; ^b^ If there are parentheses around the value, it means the GI_50_ value is displayed.

**Table 3:** Marine bioactive cyclic peptides from fungi.

Compound	Source	Mechanism	Cell Lines	IC_50_/(GI_50_) ^b^	Reference
Zygosporamide	*Zygosporium masonii*	Cytotoxicity ^a^	SF-268;	(6.5 nM)	[96]
			RXF 393	(5.0 nM)	
Cordyheptapeptide C	*Acremonium persicinum*	Cytotoxicity ^a^	SF-268;	3.7 μM;	[97]
			MCF-7;	3.0 μM;	
			NCI-H460	11.6 μM	
Cordyheptapeptide D	*Acremonium persicinum*	Cytotoxicity ^a^	SF-268;	45.6 μM;	
			MCF-7;	82.7 μM;	
			NCI-H460	>100 μM	
Cordyheptapeptide E	*Acremonium persicinum*	Cytotoxicity ^a^	SF-268;	3.2 μM;	
			MCF-7;	2.7 μM;	
			NCI-H460	4.5 μM	
Asperterrestide A	*Aspergillus terreus*	Cytotoxicity ^a^	U937;	6.4 μM;	[98]
			MOLT4	6.2 μM	
Sansalvamide A	*Microsporum* cf. *gypseum*	Inhibiting cell growth, and proliferation, and inducing cell apoptosis by regulating the expression of HSP90	HCT-116;	1.5 µM;	[99,100]
			HCT-15	1 µM	
Trapoxin	Fungal product the culture broth of *Helicoma ambiens* RF-1023	Inhibiting HDAC	NIH3T3	200 ng/mL	[104,105]
Microsporin A	*Microsporum* cf. *gypseum*	Inhibiting HDAC	HCT-116	0.6 mg/mL;	[106]
Microsporin B			HCT-116	8.5 mg/mL	

Notes: ^a^ Mechanism is yet to be investigated; ^b^ If there are parentheses around the value, it means the GI_50_ value is displayed.

**Table 4:** Marine bioactive cyclic peptides from Bacteria.

Compound	Source	Mechanism	Cell Lines	IC_50_	Reference
Mixirin A	*Bacillus species*.	Cytotoxicity ^a^	HCT-116	0.65 µM;	[108]
Mixirin B			HCT-116	1.6 µM;	
Mixirin C			HCT-116	1.26 µM;	
Mechercharmycin A	*Thermoactinomyces* sp. YM3-251	Cytotoxicity ^a^	A549;	40 nM;	[109]
			Jurkat	46 nM;	
Urukthapelstatin A	Thermoactinomycetaceae bacterium *Mechercharimyces asporophorigenens* YM11-542	Cytotoxicity ^a^	A549	12 nM;	[110]
Arenamide A	*Salinispora Arenicola*.	Inhibiting NF kappa B	293/NF-κB-Luc	3.7 μM;	[111]
Arenamide B			293/NF-κB-Luc	1.7 μM	

Notes: ^a^ Mechanism is yet to be investigated.

**Table 5:** Marine bioactive cyclic peptides from Cyanobacterial metabolites.

Compound	Source	Mechanism	Cell Lines/Target protein	IC_50_	Reference
Coibamide A	*Leptolyngbya* cyanobacterium	Cytotoxicity ^a^	NCI-H460	<23 nM	[112]
Samoamide A	*Symploca* sp.	Cytotoxicity ^a^	H460;	1.1 μM;	[113,114]
			H116	4.5 μM	
Lagunamide A	cyanobacterium, *Lyngbya majuscule,*	Cytotoxicity; Caspase-mediated mitochondrial apoptosis	A549	7.1 nM;	[116]
Lagunamide D			A549	6.7 nM	
Apratoxin A	Cyanobacterial metabolite	Down-regulating receptors and growth factor ligands for cancer cells that rely on autocrine loop	HCT-116	5.97 nM	[120]
Lyngyabellin A	cyanobacterium *Lyngbya majuscula*	Antiproliferation; Disruption of cellular microfilament network cytokinesis	KB	0.03 µg/mL;	[122]
			LoVo	0.50 µg/mL	
			Chymotrypsin	0.234 µM	
Symplocamide A	Cyanobacteria *Symploca* sp.	Inhibiting Protease	H-460;	40 nM	[123]
			Neuro-2a neuroblastoma	29 nM	
Largazole	cyanobacteria *Symploca* sp.	Inhibiting HDAC	HCT-116	44 nM	[124,125]
			Inhibit HDAC1	25 nM	

Notes: ^a^ Mechanism is yet to be investigated.

## Data Availability

Not applicable.

## Supplementary Materials

The following are available online at https://www.mdpi.com/1660-3397/19/2/115/s1, Table S1: Clinical information on Marine small peptide drugs.

## List of Abbreviations

**ADCs**	Antibody–drug conjugates
**A549**	Human non-small cell lung cancer cells
**AGS**	Human gastric cancer adenocytes cells
**APD3**	Database of antimicrobial peptides
**A-10**	Rat aortic smooth muscle cells
**AsPC-1**	Human metastatic pancreatic cancer cells
**AVPdb**	Database of antiviral peptides
**BACE1**	Beta-secretase 1
**BEL-7404**	Human liver cancer cells
**BxPC-3**	Human in situ adenocarcinoma cells
**CAR-T**	Chimeric Antigen Receptor T-Cell Immunotherapy
**CancerPPD**	Database of anticancer peptides and proteins
**Cath D**	Cathepsin D
**Cath E**	Cathepsin E
**CDX-011**	Glembatumumab vedotin
**CV1**	African green monkey kidney cells
**DLD-1**	Human colonic adenocarcinoma cells
**DU-145**	Human prostate cancer cells
**FDA**	United States Food and Drug Administration
**5-FU**	5-Fluorouracil
**GI_50_**	Half-maximal growth inhibitory concentration
**GPNMB**	Glycoprotein NMB
**HCT 8**	Human cecal adenocarcinoma cell
**HCT-116**	Human colon cancer cells
**HDAC**	Histone Deacetylase
**HeLa**	Human cervical cancer cells
**Hep G2**	Human liver cancer cells
**H460**	Human non-small cell lung cancer cells
**H-1299**	Human non-small cell lung cancer cells
**HSP90A**	Heat Shock Protein 90 Alpha Family Class A
**HT-29**	Human colon cancer cells
**IC_50_**	Half-maximal inhibitory concentration
**Jurkat 16**	T-cell leukemia cells
**JFCR39**	A panel of 39 human cancer cell lines
**KB**	Human nasopharyngeal cancer cells
**KDC**	Knottin peptide–drug conjugate
**LNCaP**	Human prostate cancer cells
**LO2**	Normal liver cells
**L1236**	Hodgkin’s Lymphoma cells
**LoVo**	Human colon cancer cells
**MCF-7**	Human breast cancer cells
**MDA-MB-231**	Human breast cancer cells
**MDR**	Multidrug resistance
**MDs**	Molecular dynamics simulation
**MMAE**	Monomethyl auristatin E
**MOLT4**	Human acute T lymphoblastic leukaemia cells
**NF-κB**	Nuclear factor kappa B
**PANC-1**	Human pancreatic cancer cells
**PC-3**	Human prostate cancer cells
**P388**	Mouse leukemia cells
**PsA**	Psammaplin A
**RBC**	Red blood cell
**ROS**	Overproduction of reactive oxygen species
**RXF 393**	Human kidney cancer cells
**SGC-7901**	Human gastric cancer cells
**SK-OV3**	Human ovarian cancer cell
**SF-268**	Human neurocancer cells
**TNF**	Tumor necrosis factor
**293/NFκB-**	Stably transfected NFκB human embryonic kidney cells
**U-87 MG**	Human glioblastoma cells
**U937**	Human histiocytic lymphoma cells
**WBC**	White blood cell
