# Supplementary material for: Recent Advances in Small Peptides of Marine Origin in Cancer Therapy
Source: Mar Drugs. 2021 Feb 19;19(2):115. doi: 10.3390/md19020115 (PMC7923226; doi:10.3390/md19020115)
Supplement: Supplementary file 1 [file marinedrugs-19-00115-s001.pdf]

Supplementary File

Recent Advances in Small Peptides of Marine Origin in Cancer Therapy

Qi-Ting Zhang [1], Ze-Dong Liu [2], Ze Wang [2], Tao Wang [1], Nan Wang [3], Ning Wang [1,*], Bin Zhang [2,*] and Yu-Fen Zhao [1]

[1] Institute of Drug Discovery Technology, Ningbo University, Ningbo, Zhejiang, 315211, PR China
[2] Li Dak Sum Yip Yio Chin Kenneth Li Marine Biopharmaceutical Research Center, Department of Marine Pharmacy, College of Food and Pharmaceutical Sciences, Ningbo University, Ningbo, Zhejiang, 315800, PR China
[3] Shenzhen Kivita Innovative Drug Discovery Institute, Shenzhen, Guangdong, 518057, PR China
* Correspondence: wangning2@nbu.edu.cn (N.W.); zhangbin1@ nbu.edu.cn (B.Z.)

Table S1. Clinical information on Marine small peptide drugs

Compound	Condition or disease	ClinicalTrials. gov Identifier:	Clinical Trial	Status	Study Completion Date
E7974	Advanced Solid Tumors	NCT00165802	**Phase I**	Completed.	January 2009
	Solid Malignancies	NCT00130169	**Phase I**	Completed.	August 2009
	Solid Malignancies	NCT00121732	**Phase I**	Completed.	October 2009
HTI-286 (Taltobulin)	Advanced solid tumours [1]	–[a]	**phase I**	Completed.	2002
Dolastatin 10 (NSC#376128)	Metastatic Prostate Cancer	NCT00003626	**Phase II**	Completed.	May 2000
	Recurrent or Metastatic Soft Tissue Sarcoma	NCT00003778	**Phase II**	Completed.	July 2000
	Refractory or Relapsed Acute Leukemia, Myelodysplastic Syndrome, or Chronic Myelogenous Leukemia	NCT00003693	Phase **I**	Completed.	October 30, 2001
	Metastatic Pancreatic Cancer	NCT00003677	**Phase II**	Completed.	January 2002
	Indolent Lymphoma, Waldenstrom's Macroglobulinemia, or Chronic Lymphocytic Leukemia	NCT00005579	**Phase II**	Completed.	February 2003
	Metastatic or Recurrent Liver, Bile Duct or Gallbladder Cancer	NCT00003557	**Phase II**	Completed.	August 2003
	Advanced Kidney Cancer	NCT00003914	**Phase II**	Completed.	May 2009
TZT-1027	Progressive Locally Advanced or Metastatic Non-Small Cell Lung Cancer	NCT00061854	**Phase II**	Completed.	May 2005
	Advanced or Metastatic Soft Tissue Sarcoma	NCT00064220	**Phase II**	Completed.	December 2005
ILX-651 (Tasidotin)	Locally Advanced or Metastatic Non-Small Cell, Lung Carcinoma Small Cell, Lung Carcinoma	NCT00078455	**Phase II**	Completed.	August 2005

	Hormone-Refractory Prostate Cancer Previously Treated with Docetaxel	NCT00082134	**Phase II**	Completed.	December 2005
	Inoperable Locally Advanced or Metastatic Melanoma	NCT00068211	**Phase II**	Completed.	January 2006
Cematodin (LU103793)	Refractory Metastatic Solid Tumors [2]	–[a]	**Phase I**	Completed.	1996
Glembatumumab vedotin (CDX-011)	Unresectable Stage III or Stage IV Melanoma	NCT00412828	**Phase I Phase II**	Completed.	May 2011
	Locally Advanced or Metastatic Breast Cancer	NCT00704158	**Phase I Phase II**	Completed.	June 2011
	Advanced GPNMB-expressing Breast Cancer	NCT01156753	**Phase II**	Completed.	November 2012
	Recurrent or Refractory Osteosarcoma	NCT02487979	**Phase II**	Completed.	June 30, 2017
	Metastatic or Locally Recurrent Uveal Melanoma	NCT02363283	**Phase II**	Completed.	July 2, 2018
	Metastatic, gpNMB Over-Expressing Triple Negative Breast Cancer	NCT01997333	**Phase II**	Completed.	August 7, 2018
	gpNMB-Expressing, Advanced or Metastatic SCC of the Lung	NCT02713828	**Phase I Phase II**	Terminated. Has Results	September 26, 2018
	Immunotherapies in Patients with Advanced Melanoma	NCT02302339	**Phase II**	Terminated. Has Results	October 3, 2018
Brentuximab vedotin (SGN-35, Monomethyl Auristatin E, Adcetris.)	A Brentuximab Vedotin Trial for Patients Who Have Previously Participated in a Brentuximab Vedotin Study	NCT00947856	**Phase II**	Completed.	March 2013
	Relapsed or Refractory Hodgkin Lymphoma	NCT00337194	**Phase II**	Completed.	October 2014
	CD30-positive Nonlymphomatous Malignancies	NCT01461538	**Phase II**	Completed.	December 2014
	Hodgkin Lymphoma	NCT00848926	**Phase II**	Completed.	May 2015
	Relapsed or Refractory Non-Hodgkin	NCT01421667	**Phase II**	Completed.	June 2015

	Lymphoma				
	Mycosis Fungoides With Variable CD30 Expression Level	NCT01396070	**Phase II**	Completed.	May 2016
	Systemic Anaplastic Large Cell Lymphoma	NCT00866047	**Phase II**	Completed.	June 2016
	Relapsed or Refractory PMLBCL (FIL_SGN01)	NCT02423291	**Phase II**	Completed.	July 2016
	Hodgkin Lymphoma	NCT01393717	**Phase II**	Completed.	February 1, 2017
	Advanced Systemic Mastocytosis or Mast Cell Leukemia	NCT01807598	**Phase II**	Completed.	September 2017
	Limited-stage Hodgkin Lymphoma	NCT01534078	**Phase II**	Completed.	January 2018
	Hodgkin Lymphoma	NCT01874054	**Phase I Phase II**	Completed.	February 20, 2018
	Relapsed or Refractory (r/r) Systemic Anaplastic Large-Cell Lymphoma or Hodgkin Lymphoma	NCT01492088	**Phase I Phase II**	Completed.	April 12, 2018
	CD30-Positive Cutaneous T-Cell Lymphoma (ALCANZA Study) (ALCANZA)	NCT01578499	**Phase III**	Completed.	July 6, 2018
	Non-bulky Limited Stage Hodgkin Lymphoma	NCT02505269	**Phase II**	Completed.	June 2019
	Relapsed/Refractory CD30-Positive Hodgkin Lymphoma (HL) or Systemic Anaplastic Large Cell Lymphoma (sALCL)	NCT02939014	**Phase II**	Completed	February 3, 2020
	High Risk of Residual Hodgkin Lymphoma Following Stem Cell Transplant (The AETHERA Trial)	NCT01100502	**Phase III**	Completed.	April 27, 2020
	CD30+ PMBL, Diffuse Large B-Cell, and Grey Zone Lymphoma Patients	NCT01994850	**Phase I Phase II**	Completed.	January 5, 2021

	Relapsed or Refractory Hodgkin Lymphoma	NCT01990534	**Phase Ⅳ**	Completed.	June 10, 2021
Didemnin B (NSC 325319)	Advanced Epithelial Ovarian Cancer [3].	–[a]	**Phase II**	Completed.	1992
	Metastatic Breast Cancer [4].	–[a]	**Phase I**	Completed.	1992
	Myeloma. A Cancer and Leukemia [5].	–[a]	**phase II**	Completed.	1994
	Advanced Malignant Melanoma [6]	–[a]	**phase II**	Completed	1998
	Central Nervous System Tumors [7]	–[a]	**Phase II**	Completed.	1999
	Recurrent or Refractory Anaplastic Astrocytoma or Glioblastoma Multiforme [8]	–[a]	**Phase II**	Completed.	1999
	Previously Treated Non-Hodgkin's lymphoma [9]	–[a]	**Phase II**	Completed	2000
Aplidine (Dehydrodidemnin B)	Advanced Prostate Cancer	NCT00780975	**Phase II**	Terminated. Has Results	March 2008
	Relapsing or Refractory Multiple Myeloma	NCT00229203	**Phase II**	Completed.	August 2008
	Relapsed/Refractory Leukemia	NCT00780143	**Phase I Phase II**	Completed.	June 2009
	Relapsed/Refractory Aggressive Non Hodgkin Lymphoma	NCT00884286	**Phase II**	Terminated. Has Results	June 2010
	Primary Myelofibrosis	NCT01149681	**Phase II**	Terminated. Has Results	February 2011
	Advanced Solid Tumors or Lymphomas	NCT00788099	**Phase I**	Completed.	June 2011
	Advanced Unresectable or Metastatic, Relapsed/Refractory, Dedifferentiated Liposarcoma (DLPS)	NCT01876043	**Phase II**	Terminated. Has Results	April 2015
	Relapsed/Refractory Myeloma (ADMYRE)	NCT01102426	**Phase III**	Completed.	November 2017
	Multiple Myeloma	NCT02100657	**Phase I**	Completed.	June 2018
	Multiple Myeloma Patients Double Refractory to Bortezomib and Lenalidomide	NCT03117361	**Phase II**	Completed.	July 2018

	Relapsed or Refractory Angioimmunoblastic T-cell Lymphoma	NCT03070964	**Phase II**	Terminated. Has Results	July 2018
	COVID-19 (APLICOV-PC)	NCT04382066	**Phase I**	Completed.	December 2020
Plinabulin	Advanced Solid Tumors or Lymphoma	NCT00322608	**Phase I**	Completed.	December 2009
	Advanced Non-Small Cell Lung Cancer	NCT00630110	**Phase I Phase II**	Completed.	June 2011
	Solid Tumors Receiving Docetaxel Myelosuppressive Chemotherapy Phase 2	NCT04345900	**Phase II**	Completed.	April 2018
	Stage IIIB-IV, Recurrent, or Metastatic Non-small Cell Lung Cancer	NCT02846792	**Phase I Phase II**	Terminated. Has Results	August 2018
	TAC Induced Neutropenia	NCT04227990	**Phase II**	Completed.	January 2019
	Advanced NSCLC (DUBLIN-3)	NCT02504489	**Phase III**	Recruiting.	November 2020
	Solid Tumors Receiving Docetaxel Myelosuppressive Chemotherapy Phase 3 (Protective-1)	NCT03102606	**Phase III**	Active, not recruiting.	December 2020
	Recurrent Small Cell Lung Cancer	NCT03575793	**Phase I Phase II**	Recruiting.	September 2022
	Metastatic Non-Small Cell Lung Cancer (NSCLC)	NCT02812667	**Phase I**	Recruiting.	December 2022
	Prevention of TAC Induced Neutropenia	NCT03294577	**Phase III**	Active, not recruiting.	September 2025
Halimide	Advanced, Metastatic Non-small Cell Lung Cancer [10]	–[a]	**Phase II**	Completed.	2015
LY355703	Inhibits the Growth of Various Tumour Cells [11]	–[a]	**Phase II**	Completed.	1998
	Phase I and pharmacological Studies of the Cryptophycin Analogue LY355703 Administered [12]	–[a]	**Phase I**	Completed.	2002

Notes: -[a] Data not mentioned.

References

1. Ratain, M.J.; Undevia, S.; Janisch, L.; Roman, S.; Mayer, P.; Buckwalter, M.; Foss, D.; Hamilton, B.L.; Fischer, J.; Bukowski, R.M. Phase I and pharmacological study of HTI-286, a novel antimicrotubule agent: correlation of neutropenia with time above threshold plasma concentration. *Proc Am Soc Clin Oncol*, **2003**,*129*, abstract 516.
2. Poruchynsky, M.S.; Kim, J.H.; Nogales, E.; Annable, T.; Loganzo, F.; Greenberger, L.M.; Sackett, D.L.; Fojo, T. Tumor cells resistant to a microtubule-depolymerizing hemiasterlin analogue, HTI-286, have mutations in alpha- or beta-tubulin and increased microtubule stability. *Biochemistry* **2004**, *43*, 13944-13954, doi:10.1021/bi049300+.
3. Cain, J.M.; Liu, P.Y.; Alberts, D.E.; Gallion, H.H.; Laufman, L.; O'Sullivan, J.; Weiss, G.; Bickers, J.N. Phase II trial of didemnin-B in advanced epithelial ovarian cancer. A Southwest Oncology Group study. *Invest New Drugs* **1992**, *10*, 23-24, doi:10.1007/bf01275473.
4. Benvenuto, J.A.; Newman, R.A.; Bignami, G.S.; Raybould, T.J.; Raber, M.N.; Esparza, L.; Walters, R.S. Phase II clinical and pharmacological study of didemnin B in patients with metastatic breast cancer. *Invest New Drugs* **1992**, *10*, 113-117, doi:10.1007/bf00873128.
5. Weiss, R.B.; Peterson, B.L.; Allen, S.L.; Browning, S.M.; Duggan, D.B.; Schiffer, C.A. A phase II trial of didemnin B in myeloma. A cancer and leukemia group B (CALGB) study. *Invest. New Drugs* **1994**, *12*, 41-43, doi:10.1007/bf00873234.
6. Hochster, H.; Oratz, R.; Ettinger, D.S.; Borden, E. A phase II study of Didemnin B (NSC 325319) in advanced malignant melanoma: an eastern cooperative oncology group study (PB687). *Invest New Drugs* **1998**, *16*, 259-263, doi:10.1023/a:1006110431250.
7. Taylor, S.A.; Giroux, D.J.; Jaeckle, K.A.; Panella, T.J.; Dakhil, S.R.; Schold, S.C. Phase II study of Didemnin B in central nervous system tumors: a southwest oncology group study. *Invest New Drugs* **1998**, *16*, 331-332, doi:10.1023/a:1006273214056.
8. Mittelman, A.; Chun, H.G.; Puccio, C.; Coombe, N.; Lansen, T.; Ahmed, T. Phase II clinical trial of didemnin B in patients with recurrent or refractory anaplastic astrocytoma or glioblastoma multiforme (NSC 325319). *Invest New Drugs* **1999**, *17*, 179-182, doi:10.1023/a:1006379402114.
9. Kucuk, O.; Young, M.L.; Habermann, T.M.; Wolf, B.C.; Jimeno, J.; Cassileth, P.A. Phase II trail of didemnin B in previously treated non-Hodgkin's lymphoma: an eastern cooperative oncology group (ECOG) study. *Am J Clin Oncol* **2000**, *23*, 273-277, doi:10.1097/00000421-200006000-00013.
10. Millward, M.; Mainwaring, P.; Mita, A.; Federico, K.; Lloyd, G.K.; Reddinger, N.; Nawrocki, S.; Mita, M.; Spear, M.A. Phase 1 study of the novel vascular disrupting agent plinabulin (NPI-2358) and docetaxel. *Invest. New Drugs* **2012**, *30*, 1065-1073, doi:10.1007/s10637-011-9642-4.
11. Mooberry, S.L.; Busquets, L.; Tien, G. Induction of apoptosis by cryptophycin 1, a new antimicrotubule agent. *Int. J. Cancer* **1997**, *73*, 440-448, doi:10.1002/(sici)1097-0215(19971104)73:3<440::aid-ijc20>3.0.co;2-f.

12. Sessa, C.; Weigang-Köhler, K.; Pagani, O.; Greim, G.; Mora, O.; De Pas, T.; Burgess, M.; Weimer, I.; Johnson, R. Phase I and pharmacological studies of the cryptophycin analogue LY355703 administered on a single intermittent or weekly schedule. *Eur. J. Cancer* **2002**, *38*, 2388-2396, doi:10.1016/s0959-8049(02)00489-6.
